# Correlation between Human Leukocyte Antigen Class II Alleles and HAI Titers Detected Post-Influenza Vaccination

**DOI:** 10.1371/journal.pone.0071376

**Published:** 2013-08-09

**Authors:** Alastair J. Moss, Fiona P. Gaughran, Aliyye Karasu, Anthony S. Gilbert, Alex J. Mann, Colin M. Gelder, John S. Oxford, Henry A. Stephens, Rob Lambkin-Williams

**Affiliations:** 1 Southampton General Hospital, Southampton, Hampshire, United Kingdom; 2 Department of Psychosis Studies, Institute of Psychiatry, King’s College, London, United Kingdom; 3 Anthony Nolan Laboratories & UCL Centre for Nephrology, Royal Free London NHS Foundation Trust, London, United Kingdom; 4 Retroscreen Virology PLC, Queen Mary BioEnterprises Innovation Centre, London, United Kingdom; 5 St. Bartholomew’s and the Royal London, Queen Mary School of Medicine and Dentistry, University of London, London, United Kingdom; Centers for Disease Control and Prevention, United States of America

## Abstract

Influenza is a major cause of morbidity and mortality. Despite vaccination, many elderly recipients do not develop a protective antibody response. To determine whether Human Leukocyte Antigen (HLA) alleles modulate seroprotection to influenza, a cohort of HLA class II-typed high-risk vaccine recipients was investigated. Haemagglutinin inhibition (HAI) titres were measured 14–40 days post-subunit vaccination. Seroprotection was defined as HAI titres reaching 40 or greater for all three vaccine strains. HLA-DRB1*04∶01 and HLA-DPB1*04∶01 alleles were detected at higher frequencies in seroprotected compared with non-seroprotected individuals. Thus, the presence of certain HLA class II alleles may determine the magnitude of antibody responses to influenza vaccination.

## Introduction

Influenza is a maor cause of morbidity and mortality worldwide with the elderly being at highest risk of complications and death. Vaccination is the main preventive strategy to minimise the impact of influenza in high risk groups. Previous meta-analyses of clinical studies in the elderly have found that vaccines well matched to the circulating influenza strain prevented up to 45% of pneumonia cases, hospital admissions and influenza-related deaths in long-term care facilities [1]. However, vaccination is not universally protective. Factors influencing the clinical effectiveness of influenza vaccines include the closeness of match to the circulating strain, previous exposure to influenza and influenza vaccines, and the immunological response mounted by the recipient [2].

Genetic polymorphisms play an important role in the immune response to viral vaccination [3]. Within the major histocompatibility complex (MHC) on chromosome 6 are genes encoding class II human leukocyte antigens (HLA), whose biological role is to present foreign peptides on the surface of antigen presenting cells to CD4+ T cells. This results in the activation, maturation and proliferation of resting CD4+ helper T cells, which induce B cells to produce antigen-specific antibody [4].There are three highly polymorphic HLA class II gene loci DR, DQ and DP, that encode allelic variants which differ in the types of antigenic peptides they preferentially bind and present to the immune system. HLA class II allele association with immune responses induced by vaccines directed against a variety of viruses including hepatitis B, measles, mumps [3] and influenza [5] have previously been described.

Influenza vaccination functions primarily by inducing influenza-specific antibodies. Antibody protection is traditionally measured by haemagglutinin-inhibition (HAI), where antibodies block the function of haemagglutinin to prevent viral attachment to host cells. HAI antibody levels closely correlate with virus neutralisation assays with serum HAI titres of 40 or greater indicative of protection [6]. Generation of HAI antibodies by B lymphocytes is regulated through CD4^+^ T cells which recognise antigens in association with HLA class II alleles. We have previously demonstrated an association between HLA class II type and non-responsiveness to influenza vaccination [5]. Our current study aims to investigate further the role between HLA class II polymorphism and seroprotection following influenza vaccination in an elderly cohort of recipients.

## Materials and Methods

### Subjects and Vaccine

185 subjects were recruited as part of the FEVER study, a randomised controlled trial (ISRCTN28553709) involving residents aged 60 years or above from 26 South London care homes [7]. In the FEVER study population, 65% of subjects were aged >80 years, 67% were female, 90% were ethnically Caucasoid and there were no Asian subjects. 70% had received influenza vaccination in 2003 and 41% had evidence of influenza vaccination prior to 2003. Residents received the 2004–5 trivalent influenza vaccine, which consisted of A/Fujian/411/2002-like (H3N2), A/New Caledonia/20/99 (H1N1) and B/Shanghai/361/2002-like virus strains. 59.3% received Influvac surface antigen, 15.6% received Fluarix split virion, 13.8% received Begrivac split virion and 11.3% received Mastaflu surface antigen. In that year the A/Fujian/411/2002-like strain was the novel component of the vaccine. Blood samples were taken 14–40 days post-vaccination. Written informed consent or assent was sought for all eligible residents. Assent for residents without capacity was requested first from the resident’s caregiver and, if unavailable, the assigned member of the care home staff [7]. Ethics approval was received from the local research ethics committee of the South London and Maudsley Trust and the Institute of Psychiatry. A greater proportion of residents with capacity participated in the trial compared to those without capacity [8].

### HAI Titres and Seroprotection

Haemagglutination (HA) was measured by the agglutination of 0.5% v/v Turkey red blood cells (TRBC’s) (Advanced Protein Products Ltd) in 96-well, v-bottomed microtitre plates (Greiner) with doubling dilutions of virus in 100 µl of Phosphate Buffered Saline (PBS) using standard protocols. Haemagglutination inhibition titres were measured using doubling dilutions of antibody and 8 Haemagglutination Units (HAU) of virus per 50 µl, again in 96-well, v-bottomed microtitre plates. Seroprotection was defined as achieving a HAI titre of 40 or greater for all three strains of the vaccine.

### Molecular HLA Class II Typing and Statistical Analysis

High quality full-length genomic DNA was prepared from blood clots using a Masterpure DNA purification kit with a modified salting out technique [9]. HLA-DRB1, DRB3, DRB4, DRB5 and DQB1 allele profiles were determined using HLA class II sequence specific oligonucleotide probes (SSOP) ligated to fluorescent labeled microspheres with the Luminex platform [10]. DPB1 alleles were determined by direct sequencing. Full 4-figure molecular HLA class II allele nomenclature was used when unambiguous types were available, while 2-figure group- specific nomenclature was used for DRB1*07, *09, *11, *12 and *14 groups. Predicted HLA class II phenotype or antigen frequencies (AgF) were determined by directed counting of identified alleles and expressed as percentage of the number of individuals in each test group using the following formula: AgF(%) = n/N×100, where n = number of individuals scoring positive for a given allele and N = total number of individuals in the test group. HLA class II AgF’s were then compared in vaccine responder and non-responder groups using the Chi-square test, P values <0.05 were considered significant, with odds ratios and confidence intervals as given. Given the relative size of the test study (185 subjects), Linkage disequilibrium exists between different HLA class II loci and therefore Bonferroni’s adjustment for multiple comparisons was omitted to avoid over-correction and the generation of false negative results. HLA class II allele frequencies (AF) were also determined for specific alleles showing significant AgF associations with vaccine response, using the following formula AF(%) = n/2N×100 (where 2N represents the total number of chromosomes studied in the test group).

## Results

### Seroprotection Following Influenza Vaccination

HAI titers of ≥40 were detected post-vaccination in 50% (n = 92) of elderly vaccine recipients to all three strains and were considered vaccine responders. Seroprotection rates to the A/Fujian/411/2002-like strain (96% of recipients; n = 177) were higher than other strains (A/New Caledonian/20/99 64%, n = 118; B/Shanghai/361/2002-like 63%, n = 116) after vaccination. In an elderly population immunosenescence may affect the response to influenza vaccine, we analysed the response in those aged 60–80 and those over 80 and found no statistically significant difference.

### HLA Class II Antigen Frequencies in Vaccine Responder Groups

Significant increases in HLA-DRB1*04∶01 AgF (Table 1) and HLA-DPB1*04∶01 AgF (Table 2) were observed in seroprotected recipients compared to the non-seroprotected group. These associations with vaccine response became stronger when the AF as a measure of the number of chromosomes carrying DRB1*04∶01(Chi-square = 6.6, P = 0.01, OR = 2.6 (1.2<OR<5.7) or DPB1*0401 (Chi-square = 6.2, P = 0.01, OR = 1.7 (1.1<OR<2.8) were calculated. There was no significant increase in frequency of any HLA-DQB1 alleles among seroprotected compared with non-seroprotected individuals (data not shown). There was an increased frequency of the combination of HLA-DRB1*04∶01 and HLA-DPB1*04∶01 alleles in the seroprotected compared with non-seroprotected individuals (Table 3). By contrast, the absence of both these alleles was significantly more common in the non-seroprotected vaccine recipients.

**Table 1:** HLA-DRB1 antigen frequencies (AgF) in vaccine responder (seroprotected) and non-responder (non-seroprotected) groups.

HLA-DRB1 alleles	Seroprotected N = 92 n = (AgF%)	Non-seroprotected N = 93 n = (AgF%)	p value	Odds ratio	95% Confidence interval
*01∶01	17 (8.5)	9 (13.0)	NS		
*01∶02	4 (4.3)	2 (2.2)	NS		
*01∶03	2 (2.2)	3 (3.2)	NS		
*03∶01	21 (22.8)	25 (25.8)	NS		
*03∶02	1 (1.1)	1 (1.1)	NS		
*04∶01	22 (23.9)	11 (11.8)	0.03	2.3	1.0–5.8
*04∶02	1 (1.1)	1 (1.1)	NS		
*04∶04	9 (9.8)	9 (9.7)	NS		
*04∶07	2 (2.2)	2 (2.2)	NS		
*04∶08	0	2 (2.2)	NS		
*07	25 (27.2)	22 (23.4)	NS		
*08∶01	5 (5.4)	3 (3.3)	NS		
*08∶02	2 (2.2)	0	NS		
*08∶03	1 (1.1)	0	NS		
*08∶04	2 (2.2)	0	NS		
*09	3 (3.3)	3 (3.3)	NS		
*10∶01	0	2 (2.2)	NS		
*11	12 (13.0)	18 (19.4)	NS		
*12	3 (3.3)	4 (20.4)	NS		
*13∶01	10 (10.9)	11 (11.8)	NS		
*13∶02	5 (5.4)	7 (7.5)	NS		
*13∶03	1 (1.1)	1 (1.1)	NS		
*14	4 (4.3)	5 (5.4)	NS		
*15∶01	18 (19.6)	25 (26.9)	NS		
*15∶02	0	2 (2.2)	NS		
*15∶03	2 (2.2)	1 (1.1)	NS		
*16.01	2 (2.2)	1 (1.1)	NS		
*16.02	1 (1.1)	1 (1.1)	NS		

Responder = HAI ≥40.

Non-responder = HAI <40.

AgF% = antigen frequency expressed as a percentage.

NS = not significant.

**Table 2:** HLA-DPB1 antigen frequencies (AgF) in vaccine responder (seroprotected) and non-responder (non-seroprotected) groups.

HLA-DPB1 alleles	Seroprotected N = 92 n = (AgF%)	Non-seroprotected N = 93 n = (AgF%)	p value	Odds ratio	95% Confidence interval
*01∶01	9 (10.3)	13 (14.7)	NS		
*02∶01	23 (26.4)	22 (25.0)	NS		
*02∶02	1 (1.1)	1 (1.1)	NS		
*03∶01	18 (20.7)	21 (23.9)	NS		
*04∶01	62 (71.2)	47 (53.4)	0.015	2.16	1.11–4.25
*04∶02	17 (19.5)	24 (27.2)	NS		
*05∶01	1 (1.1)	4 (4.5)	NS		
*06∶01	1 (1.1)	2 (2.2)	NS		
*10∶01	2 (2.2)	4 (4.5)	NS		
*11∶01	5 (5.7)	3 (4.2)	NS		
*13∶01	5 (5.7)	3 (3.4)	NS		
*14∶01	1 (1.1)	3 (3.4)	NS		
*15∶01	0	1 (1.1)	NS		
*16∶01	0	1 (1.1)	NS		
*17∶01	3 (3.4)	3	NS		
*19∶01	1 (1.1)	0	NS		
*20∶01	0	1 (1.4)	NS		
*21∶01	0	1 (1.1)	NS		
*23∶01	0	1 (1.1)	NS		
*85.01	1 (1.1)	0	NS		

Responder = HAI ≥40.

Non-responder = HAI <40.

AgF% = antigen frequency expressed as a percentage.

NS = not significant.

**Table 3:** Frequency of HLA-DRB1*0401 and HLA-DPB1*0401 combinations in vaccine responder (seroprotected) and non-responder (non-seroprotected) groups.

HLA allele combinations	Seroprotected N = 87 n = (AgF%)	Non-seroprotected N = 88 n = (AgF%)	p value	Odds ratio	95% Confidence intervaI
DRB1*04∶01+ve/DPB1*04∶01+ve	16 (18.4)*A	7 (7.9)*A	0.041	2.61	0.94–7.5
DRB1*04∶01+ve/DPB1*04∶01–ve	4 (4.5)	4 (4.5)			
DRB1*04∶01–ve/DPB1*04∶01+ve	46 (52.8)	40 (45.4)			
DRB1*04∶01–ve/DPB1*04∶01–ve	21 (24.1)*B	37 (42.0)*B	0.012	2.28	1.14–4.6

Responder = HAI ≥40.

Non-responder = HAI <40.

Combined Chi-square = 8.35; 3 degrees of freedom; P value = 0.04.

* A = Chi-square = 4.2; *B = Chi-square = 6.3.

## Discussion

HLA allele polymorphisms are associated with variations in immune response to several viral vaccines [3]. However, there is a relative paucity of information regarding seasonal influenza vaccination. We have previously described an HLA class II association with non-responsiveness to influenza vaccination, where individuals with HLA-DRB1*07 produced lower antibody titres following a single administration of influenza vaccine [5]. A limited number of influenza viral peptides generate a focused CD4^+^ and CD8^+^ response, which is termed immune-dominance. Peptide binding to HLA alleles is a major limiting step in generating this targeted immune response. Hydrogen binding between influenza peptides and certain HLA alleles allows more prominent, solvent-exposed structures within the peptide to generate an effective T cell response [3].

The production of IgG antibodies by B Cells requires CD4+ T cell help and thus, MHC presentation of viral peptides to CD4+ T cells is an important step in mediating the adaptive immune response following vaccination. In our study HLA-DRB1*04∶01 and HLA-DPB1*04∶01 occurred at higher frequencies in individuals with seroprotective levels of haemagglutinin antibody. These alleles are encoded by distinct HLA class II loci, but are relatively common in Caucasoid populations (see www.allelefrequencies.net) and share some sequence similarities in regions of their antigen binding grooves thought to be critical in peptide binding (pos 65–75, see www.ebi.ac.uk/imgt/hla). Haemagglutinin peptide is highly immunogenic and can induce CD4+ T cell responses against conserved and variable regions [5]. There is clear evidence that influenza haemagglutinin peptide HA 307–319 interacts with T cell receptors when displayed by HLA-DRB1*04∶01 [11]. Moreover, DRB1*04∶01 tetramers loaded with HA 306–318 have demonstrated increased frequency of CD4+ T Cells-restricted to HA 306–318 following influenza vaccination [12]. Recent tetramer guided epitope mapping for H5N1 in mouse models have identified H5HA 57–76 and H5HA 441–460 to be immunodominant epitopes for DRB1*04∶01 [13]. Hence individuals with allele HLA-DR4 may more easily facilitate haemagglutinin processing cumulating in higher antibody titres. Interestingly, immune responses to hepatitis B vaccine are positively associated with HLA-DPB1*04∶01 which may suggest that these alleles are involved in a generic response to vaccination [14]. However, HLA-DRB1*04∶01 has been found to be associated with non-responder status to hepatitis B vaccine in US Caucasoids [15].

Following vaccination, almost all recipients in our study had aseroprotective level of HAI against influenza A/Fujian and yet only half of the cohort was universally seroprotected against all three strains. Interestingly, Ohmit et al. [16] have noted that live-attenuated and inactivated vaccine recipients who had HAI titres above a seroprotective level may still be at risk of vaccine failure. The immune response to influenza vaccination is clearly very complex and the reason individuals survive to old age in the absence of seroprotective antibody responses remains unclear. A number of postulates may account for the failure to mount an appropriate antibody response in the elderly. McElhaney et al. [17] highlighted that whilst antibody responses are a surrogate marker for vaccine efficacy in the elderly, cytokine production (IFN-gamma: IL-10 ratio, granzyme B levels) may serve as a marker of cell-mediated immune response. In addition, these age-related changes in cytokine dynamics may reflect changes in innate immunity.

The immune response to influenza vaccination is complex requiring adequate antigen recognition to initiate T cell activation [18]. Allelic variants of HLA class II molecules, through their role in antigen presentation to CD4+ T helper cells, most probably influence the adequacy of the immune response following influenza vaccination. Our study demonstrates a significant association between two relatively common HLA class II alleles (DRB1*04∶01 and DPB1*04∶01) with a higher seroprotective response to trivalent seasonal influenza vaccination in an elderly cohort, and warrants further investigation to validate these observations. Nevertheless, future influenza vaccine trials may need to account for the genetic variability of the target population when evaluating efficacy of vaccine response in elderly or other immuno-compromised and vulnerable groups.
